# *catena*-Poly[[silver(I)-μ-4,4′-bipyridine-κ^2^ *N*:*N*′] 4-[2-(4-carb­oxy­phen­yl)-1,1,1,3,3,3-hexa­fluoro­propan-2-yl]benzoate]

**DOI:** 10.1107/S1600536812015322

**Published:** 2012-04-18

**Authors:** Xiu-Juan Jiang

**Affiliations:** a College of Biological, Chemical Sciences and Engineering, Jiaxing University, Zhejiang Jiaxing 314001, People’s Republic of China

## Abstract

Assembly of the flexible dicarb­oxy­lic ligand 4-[2-(4-carboxyphenyl)-1,1,1,3,3,3-hexafluoropropan-2-yl]benzoate and 4,4′-bipyridine as co-ligand with Ag^I^ ions resulted in the formation of the polymeric title compound, {[Ag(C_10_H_8_N_2_)](C_17_H_9_F_6_O_4_)}_*n*_, in which the metal atoms are bridged by the 4,4′-bipyridine ligands, generating cationic chains extending along [010]. The dihedral angles between the benzene rings in the anion and the pyridine rings in the cation are 72.42 (9) and 9.36 (10)°, respectively. The mol­ecular conformation of the anion is stabilized by intra­molecular C—H⋯F hydrogen bonds. In the crystal, the anions inter­act with the cationic chains *via* C—H⋯O hydrogen bonds, forming layers parallel to (001), in which weak π–π stacking inter­actions [centroid–centroid distances = 3.975 (3)–4.047 (3) Å] involving the pyridine rings of adjacent 4,4′-bipyridine ligands are present. The planes are further assembled into a three-dimensional network by O—H⋯O hydrogen bonds.

## Related literature

For background to metal-organic frameworks, see: Du *et al.* (2007 ▶); Li & Du (2011 ▶); Hosseini (2005 ▶). For metallosupra­molecular architectures, see: Brammer (2004 ▶); Peedikakkal & Vittal (2011 ▶). For coordination frameworks constructed from pyridyl and carboxyl­ate spacers, see: Li *et al.* (2012 ▶). For weak cooperative inter­molecular inter­actions, see: Ye *et al.* (2005 ▶). For flexible polycarboxyl ligands, see: Liu *et al.* (2011 ▶). For the structures of metal complexes derived from 4-[2-(4-carboxy­phenyl)-1,1,1,3,3,3-hexafluoropropan-2-yl]benzoate, see: Jiang *et al.* (2009 ▶); Ji *et al.* (2010 ▶).
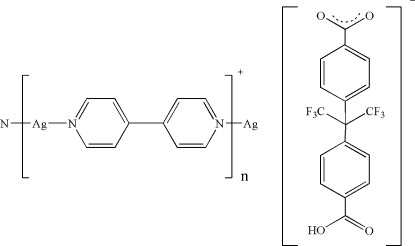


## Experimental

### 

#### Crystal data

[Ag(C_10_H_8_N_2_)](C_17_H_9_F_6_O_4_)
*M* *_r_* = 655.30
Monoclinic, 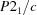

*a* = 16.434 (7) Å
*b* = 11.436 (5) Å
*c* = 14.320 (6) Å
β = 104.310 (7)°
*V* = 2607.9 (19) Å^3^
*Z* = 4
Mo *K*α radiation
μ = 0.85 mm^−1^
*T* = 296 K
0.24 × 0.20 × 0.18 mm

#### Data collection

Bruker APEXII CCD area-detector diffractometer
Absorption correction: multi-scan (*SADABS*; Bruker, 2001 ▶) *T*_min_ = 0.817, *T*_max_ = 0.862
12604 measured reflections
4604 independent reflections
3593 reflections with *I* > 2σ(*I*)
*R*_int_ = 0.029

#### Refinement

*R*[*F* ^2^ > 2σ(*F* ^2^)] = 0.033
*wR*(*F* ^2^) = 0.080
*S* = 1.06
4604 reflections
362 parameters
H-atom parameters constrained
Δρ_max_ = 0.32 e Å^−3^
Δρ_min_ = −0.47 e Å^−3^

### 

Data collection: *APEX2* (Bruker, 2003 ▶); cell refinement: *SAINT* (Bruker, 2001 ▶); data reduction: *SAINT*; program(s) used to solve structure: *SHELXS97* (Sheldrick, 2008 ▶); program(s) used to refine structure: *SHELXL97* (Sheldrick, 2008 ▶); molecular graphics: *SHELXTL* (Sheldrick, 2008 ▶); software used to prepare material for publication: *SHELXTL*.

## Supplementary Material

Crystal structure: contains datablock(s) I, global. DOI: 10.1107/S1600536812015322/rz2731sup1.cif

Structure factors: contains datablock(s) I. DOI: 10.1107/S1600536812015322/rz2731Isup2.hkl

Additional supplementary materials:  crystallographic information; 3D view; checkCIF report

## Figures and Tables

**Table 1:** Hydrogen-bond geometry (Å, °)

*D*—H⋯*A*	*D*—H	H⋯*A*	*D*⋯*A*	*D*—H⋯*A*
O4—H4⋯O2^i^	0.82	1.72	2.538 (2)	174
C4—H4*A*⋯O1^ii^	0.93	2.52	3.366 (4)	152
C7—H7⋯O1^ii^	0.93	2.54	3.297 (4)	138
C8—H8⋯O3	0.93	2.53	3.312 (4)	142
C9—H9⋯O2^iii^	0.93	2.48	3.242 (4)	139
C16—H16⋯F4	0.93	2.35	2.992 (4)	126
C26—H26⋯F3	0.93	2.33	2.941 (4)	123

Symmetry codes: (i) 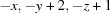; (ii) 
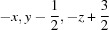
; (iii) 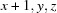.

**Fig. 1.**
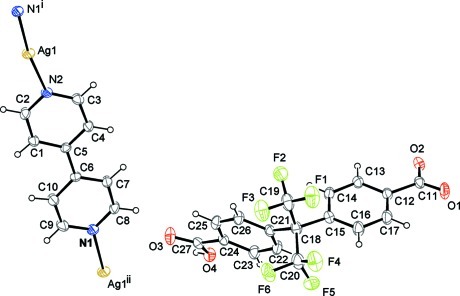
The asymmetric unit of title compound with 30% probability displacement ellipsoids [symmetry codes: (i) x, -1+y, z; (ii) = x, 1+y,z).

**Fig. 2.**
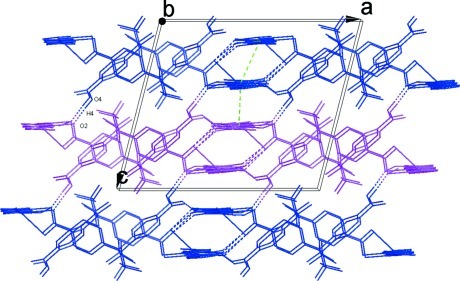
View of the 3-D supramolecular organization of the title compound constructed from π–π stacking interactions (green dotted lines) and hydrogen bonds (dotted lines). Overlapping layers parallel to the ab plane are coloured in blue and magenta.

## supplementary crystallographic information

### Comment

Research on metal-organic frameworks (MOFs) has been rapidly developed to produce new materials with fascinating structural topologies and potential applications (Du *et al.*, 2007; Li & Du, 2011; Hosseini *et al.*, 2005). Generally, the diversity of the network structures of such materials greatly depends on the selection of the well designed organic ligands and metal centers, and a variety of novel metallosupramolecular architectures have been obtained so far (Brammer *et al.*, 2004; Peedikakkal *et al.*, 2011). Accordingly, ligands with certain functional groups, such as pyridyl and carboxylate have been widely explored to construct such coordination frameworks (Li *et al.*, 2012). In addition, aside from the fundamental coordination driven force, other weak cooperative intermolecular interactions such as hydrogen bonding and aromatic stacking are also useful tools in the construction of such crystalline solids (Ye *et al.*, 2005). Among the versatile dicarboxylate tectons, the conformational freedom nature of the flexible polycarboxyl modules may provide more possibility for the construction of unusual coordination frameworks compared with rigid ligands (Liu *et al.*, 2011). 4,4'-(Hexafluoroisopropylidene)bis(benzoic acid) (H_2_L) as a V-shaped flexible dicarboxyl ligand has been investigated for its bent geometry, which can induce novel topological motifs and potential functional materials (Jiang *et al.*, 2009; Ji *et al.*, 2010). Herein the synthesis and structure of a new crystalline complex is reported, generated from the incorporation of the V-shaped dicarboxyl spacer H_2_L with 4,4'-bipyridine (bipy) as co-ligand and Ag^I^ ion, which displays a one-dimensional polymeric chain motif.

The asymmetric unit of the title compound consists of one silver(I) cation, one bipy molecule and one HL anion (Fig. 1). Each metal atom is coordinated in a nearly linear geometry (N2—Ag1—N1^i^ = 171.36 (9)°; symmetry code: (i) x, -1+y, z) by a pair of nitrogen donors from two bipy molecules, forming one-dimensional polymeric chain motifs along the [010] direction with Ag···Ag separations of 11.436 (5) Å. If the Ag···O separations with the monoprotonated carboxylate anion are considered (Ag1···O2, 2.680 (2) Å; Ag1···O1, 2.701 (2) Å), the HL ligands can be seen as pendants of the Ag-bipy chains. In the cation, the dihedral angle formed by the pyridine rings is 72.42 (9)°, while the dihedral angle formed by the benzene rings in the anion is 9.36 (10)°. The molecular conformation of the anion is enforced by intramolecular C—H···F hydrogen bonds (Table 1). In the crystal packing, anions and cationic chains interact through C—H···O hydrogen bonds to generate layers parallel to the *ab* plane. In each layer, weak π–π stacking interactions (centroid-to-centroid distances = 3.975 (3)–4.047 (3) Å) involving the pyridine rings of adjacent bipy ligands are observed (Fig. 2). Interplanar O—H···O hydrogen bond involving the carboxylic and carboxylate groups further assemble the layers in a three-dimensional network.

### Experimental

A mixture of H_2_L (20.2 mg, 0.05 mmol), silver acetate (16.8 mg, 0.10 mmol), 4,4'-bipyridine (15.4 mg, 0.10 mmol) and water (10 ml) was sealed in a Teflon-lined stainless steel vessel (20 ml), which was heated at 433°C for three days and then cooled to room temperature. Colourless needle-like crystals of title compound were obtained in 61% yield (20.0 mg, based on H_2_L).

### Refinement

The carbonyl H atom was first located in a difference Fourier map and then refined with O—H = 0.82 Å, and with *U*_iso_(H) = 1.5*U*_eq_(O). The C-bound H atoms were placed at calculated positions and refined as riding with C—H = 0.93 Å, and with *U*_iso_(H) = 1.2*U*_eq_(C).

### Crystal data

**Table:**

[Ag(C_10_H_8_N_2_)](C_17_H_9_F_6_O_4_)	*F*(000) = 1304
*M**_r_* = 655.30	*D*_x_ = 1.669 Mg m^−^^3^
Monoclinic, *P*2_1_/*c*	Mo *K*α radiation, λ = 0.71073 Å
Hall symbol: -P 2ybc	Cell parameters from 3592 reflections
*a* = 16.434 (7) Å	θ = 2.6–25.0°
*b* = 11.436 (5) Å	µ = 0.85 mm^−^^1^
*c* = 14.320 (6) Å	*T* = 296 K
β = 104.310 (7)°	Block, colourless
*V* = 2607.9 (19) Å^3^	0.24 × 0.20 × 0.18 mm
*Z* = 4	

### Data collection

**Table:**

Bruker APEXII CCD area-detector diffractometer	4604 independent reflections
Radiation source: fine-focus sealed tube	3593 reflections with *I* > 2σ(*I*)
Graphite monochromator	*R*_int_ = 0.029
phi and ω scans	θ_max_ = 25.0°, θ_min_ = 1.3°
Absorption correction: multi-scan (*SADABS*; Bruker, 2001)	*h* = −19→18
*T*_min_ = 0.817, *T*_max_ = 0.862	*k* = −13→11
12604 measured reflections	*l* = −13→17

### Refinement

**Table:**

Refinement on *F*^2^	Primary atom site location: structure-invariant direct methods
Least-squares matrix: full	Secondary atom site location: difference Fourier map
*R*[*F*^2^ > 2σ(*F*^2^)] = 0.033	Hydrogen site location: inferred from neighbouring sites
*wR*(*F*^2^) = 0.080	H-atom parameters constrained
*S* = 1.06	*w* = 1/[σ^2^(*F*_o_^2^) + (0.0345*P*)^2^ + 0.8598*P*] where *P* = (*F*_o_^2^ + 2*F*_c_^2^)/3
4604 reflections	(Δ/σ)_max_ = 0.001
362 parameters	Δρ_max_ = 0.32 e Å^−^^3^
0 restraints	Δρ_min_ = −0.47 e Å^−^^3^

### Special details

**Table:**

Geometry. All e.s.d.'s (except the e.s.d. in the dihedral angle between two l.s. planes) are estimated using the full covariance matrix. The cell e.s.d.'s are taken into account individually in the estimation of e.s.d.'s in distances, angles and torsion angles; correlations between e.s.d.'s in cell parameters are only used when they are defined by crystal symmetry. An approximate (isotropic) treatment of cell e.s.d.'s is used for estimating e.s.d.'s involving l.s. planes.
Refinement. Refinement of *F*^2^ against ALL reflections. The weighted *R*-factor *wR* and goodness of fit *S* are based on *F*^2^, conventional *R*-factors *R* are based on *F*, with *F* set to zero for negative *F*^2^. The threshold expression of *F*^2^ > σ(*F*^2^) is used only for calculating *R*-factors(gt) *etc*. and is not relevant to the choice of reflections for refinement. *R*-factors based on *F*^2^ are statistically about twice as large as those based on *F*, and *R*- factors based on ALL data will be even larger.

### Fractional atomic coordinates and isotropic or equivalent isotropic displacement parameters (Å^2^)

**Table:**

	*x*	*y*	*z*	*U*_iso_*/*U*_eq_	
Ag1	0.530651 (16)	−0.082641 (18)	0.622126 (19)	0.04658 (10)	
F1	0.02974 (15)	0.6454 (2)	0.92201 (18)	0.0909 (8)	
F2	0.06031 (13)	0.61728 (17)	0.78671 (18)	0.0684 (6)	
F3	0.15860 (14)	0.6401 (2)	0.91554 (16)	0.0829 (7)	
F4	0.09383 (15)	0.8354 (2)	1.01974 (14)	0.0868 (8)	
F5	0.10424 (14)	0.9881 (2)	0.93702 (15)	0.0757 (7)	
F6	0.20357 (13)	0.8623 (3)	0.96704 (15)	0.0886 (8)	
O1	−0.32135 (14)	0.9301 (2)	0.75562 (17)	0.0601 (7)	
O2	−0.31043 (12)	0.90448 (18)	0.60650 (15)	0.0447 (5)	
O3	0.33356 (15)	0.9023 (2)	0.56863 (18)	0.0608 (7)	
O4	0.24921 (14)	1.05711 (19)	0.53684 (16)	0.0484 (6)	
H4	0.2713	1.0657	0.4919	0.073*	
N1	0.52282 (16)	0.7266 (2)	0.62575 (17)	0.0404 (6)	
N2	0.51817 (15)	0.1071 (2)	0.61686 (17)	0.0368 (6)	
C1	0.58883 (19)	0.2880 (2)	0.6165 (2)	0.0423 (8)	
H1	0.6375	0.3270	0.6123	0.051*	
C2	0.5861 (2)	0.1688 (2)	0.6116 (2)	0.0424 (8)	
H2	0.6334	0.1288	0.6043	0.051*	
C3	0.4518 (2)	0.1674 (3)	0.6274 (2)	0.0449 (8)	
H3	0.4039	0.1262	0.6306	0.054*	
C4	0.45017 (19)	0.2870 (2)	0.6337 (2)	0.0412 (8)	
H4A	0.4023	0.3247	0.6421	0.049*	
C5	0.52023 (17)	0.3515 (2)	0.62763 (19)	0.0314 (7)	
C6	0.52118 (18)	0.4813 (2)	0.63058 (19)	0.0331 (7)	
C7	0.44988 (19)	0.5469 (2)	0.6263 (2)	0.0398 (7)	
H7	0.3993	0.5096	0.6251	0.048*	
C8	0.4531 (2)	0.6668 (2)	0.6239 (2)	0.0416 (8)	
H8	0.4038	0.7083	0.6207	0.050*	
C9	0.5923 (2)	0.6642 (3)	0.6312 (3)	0.0568 (10)	
H9	0.6421	0.7040	0.6331	0.068*	
C10	0.5941 (2)	0.5440 (3)	0.6341 (3)	0.0545 (9)	
H10	0.6445	0.5046	0.6385	0.065*	
C11	−0.27936 (19)	0.9090 (2)	0.6968 (2)	0.0399 (7)	
C12	−0.18662 (18)	0.8837 (3)	0.7344 (2)	0.0385 (7)	
C13	−0.13701 (19)	0.8418 (3)	0.6766 (2)	0.0410 (7)	
H13	−0.1609	0.8275	0.6117	0.049*	
C14	−0.05230 (19)	0.8210 (3)	0.7143 (2)	0.0430 (8)	
H14	−0.0199	0.7935	0.6741	0.052*	
C15	−0.01503 (19)	0.8405 (3)	0.8103 (2)	0.0396 (7)	
C16	−0.0646 (2)	0.8806 (3)	0.8685 (2)	0.0558 (9)	
H16	−0.0410	0.8934	0.9337	0.067*	
C17	−0.1491 (2)	0.9019 (3)	0.8306 (2)	0.0538 (9)	
H17	−0.1814	0.9291	0.8710	0.065*	
C18	0.07922 (19)	0.8103 (3)	0.8496 (2)	0.0434 (8)	
C19	0.0819 (2)	0.6771 (4)	0.8687 (3)	0.0611 (10)	
C20	0.1197 (2)	0.8734 (4)	0.9440 (3)	0.0638 (10)	
C21	0.13085 (18)	0.8446 (3)	0.7768 (2)	0.0389 (7)	
C22	0.1201 (2)	0.9558 (3)	0.7381 (2)	0.0479 (8)	
H22	0.0809	1.0055	0.7542	0.057*	
C23	0.1665 (2)	0.9947 (3)	0.6759 (2)	0.0472 (8)	
H23	0.1586	1.0702	0.6512	0.057*	
C24	0.22482 (18)	0.9221 (3)	0.6502 (2)	0.0387 (7)	
C25	0.2352 (2)	0.8112 (3)	0.6884 (2)	0.0461 (8)	
H25	0.2740	0.7615	0.6716	0.055*	
C26	0.18943 (19)	0.7715 (3)	0.7511 (2)	0.0452 (8)	
H26	0.1978	0.6962	0.7761	0.054*	
C27	0.27476 (19)	0.9585 (3)	0.5814 (2)	0.0408 (7)	

### Atomic displacement parameters (Å^2^)

**Table:**

	*U*^11^	*U*^22^	*U*^33^	*U*^12^	*U*^13^	*U*^23^
Ag1	0.04784 (17)	0.02023 (13)	0.06987 (19)	0.00120 (10)	0.01111 (12)	−0.00107 (11)
F1	0.0822 (18)	0.0897 (17)	0.116 (2)	0.0119 (14)	0.0525 (16)	0.0463 (15)
F2	0.0601 (14)	0.0440 (11)	0.1002 (17)	0.0030 (10)	0.0179 (12)	0.0053 (12)
F3	0.0614 (15)	0.0972 (17)	0.0862 (16)	0.0294 (13)	0.0110 (12)	0.0452 (14)
F4	0.0702 (16)	0.150 (2)	0.0394 (12)	0.0082 (15)	0.0126 (11)	0.0089 (13)
F5	0.0730 (16)	0.0876 (17)	0.0653 (14)	−0.0097 (13)	0.0145 (11)	−0.0295 (13)
F6	0.0400 (13)	0.155 (2)	0.0620 (13)	0.0041 (14)	−0.0043 (10)	−0.0173 (15)
O1	0.0368 (14)	0.0868 (19)	0.0628 (15)	0.0103 (12)	0.0241 (12)	−0.0029 (13)
O2	0.0298 (12)	0.0521 (14)	0.0524 (14)	−0.0024 (10)	0.0106 (10)	0.0057 (11)
O3	0.0441 (15)	0.0664 (17)	0.0788 (17)	0.0175 (12)	0.0285 (13)	0.0164 (13)
O4	0.0456 (14)	0.0492 (13)	0.0532 (14)	0.0041 (11)	0.0173 (11)	0.0054 (11)
N1	0.0398 (16)	0.0243 (13)	0.0561 (16)	0.0021 (11)	0.0095 (12)	−0.0007 (11)
N2	0.0363 (16)	0.0247 (13)	0.0467 (15)	0.0017 (10)	0.0050 (12)	−0.0012 (10)
C1	0.0346 (18)	0.0278 (15)	0.066 (2)	−0.0027 (13)	0.0154 (16)	0.0025 (14)
C2	0.0369 (19)	0.0289 (15)	0.064 (2)	0.0056 (13)	0.0182 (16)	0.0020 (15)
C3	0.038 (2)	0.0294 (16)	0.066 (2)	−0.0050 (14)	0.0113 (16)	0.0012 (15)
C4	0.0319 (18)	0.0280 (15)	0.065 (2)	0.0019 (12)	0.0147 (15)	0.0002 (14)
C5	0.0338 (18)	0.0251 (15)	0.0343 (15)	0.0030 (12)	0.0064 (13)	0.0000 (12)
C6	0.0368 (18)	0.0236 (15)	0.0393 (17)	−0.0010 (12)	0.0102 (13)	−0.0002 (12)
C7	0.0333 (18)	0.0265 (15)	0.063 (2)	0.0005 (12)	0.0177 (15)	0.0014 (14)
C8	0.038 (2)	0.0272 (15)	0.063 (2)	0.0080 (13)	0.0193 (16)	0.0023 (14)
C9	0.038 (2)	0.0297 (17)	0.101 (3)	−0.0049 (14)	0.0131 (19)	−0.0053 (18)
C10	0.0336 (19)	0.0281 (16)	0.100 (3)	0.0026 (14)	0.0124 (18)	−0.0043 (17)
C11	0.0321 (17)	0.0323 (16)	0.057 (2)	−0.0007 (13)	0.0138 (16)	0.0050 (15)
C12	0.0308 (17)	0.0368 (16)	0.0505 (19)	−0.0014 (13)	0.0150 (14)	0.0009 (14)
C13	0.0338 (18)	0.0466 (18)	0.0408 (17)	0.0034 (14)	0.0060 (14)	−0.0029 (15)
C14	0.0350 (19)	0.0513 (19)	0.0442 (18)	0.0081 (15)	0.0129 (15)	−0.0053 (15)
C15	0.0303 (18)	0.0459 (18)	0.0423 (18)	0.0006 (14)	0.0087 (14)	0.0027 (14)
C16	0.040 (2)	0.088 (3)	0.0399 (19)	0.0051 (19)	0.0112 (15)	−0.0084 (18)
C17	0.038 (2)	0.077 (3)	0.051 (2)	0.0028 (18)	0.0194 (16)	−0.0100 (18)
C18	0.0349 (19)	0.055 (2)	0.0395 (18)	0.0039 (15)	0.0073 (14)	0.0052 (15)
C19	0.045 (2)	0.069 (3)	0.071 (3)	0.0093 (19)	0.018 (2)	0.027 (2)
C20	0.043 (2)	0.098 (3)	0.049 (2)	0.006 (2)	0.0063 (18)	−0.004 (2)
C21	0.0263 (17)	0.0460 (18)	0.0414 (17)	0.0026 (13)	0.0028 (13)	0.0007 (14)
C22	0.041 (2)	0.0462 (19)	0.062 (2)	0.0112 (15)	0.0232 (17)	0.0015 (16)
C23	0.042 (2)	0.0393 (18)	0.063 (2)	0.0063 (15)	0.0182 (17)	0.0059 (16)
C24	0.0271 (16)	0.0458 (18)	0.0406 (17)	0.0006 (14)	0.0033 (13)	−0.0029 (15)
C25	0.039 (2)	0.049 (2)	0.052 (2)	0.0141 (15)	0.0130 (16)	0.0012 (16)
C26	0.0374 (19)	0.0458 (19)	0.052 (2)	0.0113 (15)	0.0110 (15)	0.0051 (15)
C27	0.0292 (18)	0.0460 (18)	0.0438 (18)	−0.0028 (14)	0.0025 (14)	−0.0025 (15)

### Geometric parameters (Å, º)

**Table:**

Ag1—N2	2.179 (2)	C8—H8	0.9300
Ag1—N1^i^	2.186 (2)	C9—C10	1.376 (4)
F1—C19	1.332 (4)	C9—H9	0.9300
F2—C19	1.329 (4)	C10—H10	0.9300
F3—C19	1.342 (4)	C11—C12	1.514 (4)
F4—C20	1.332 (4)	C12—C17	1.380 (4)
F5—C20	1.335 (5)	C12—C13	1.383 (4)
F6—C20	1.341 (4)	C13—C14	1.384 (4)
O1—C11	1.238 (4)	C13—H13	0.9300
O2—C11	1.268 (4)	C14—C15	1.378 (4)
O3—C27	1.212 (4)	C14—H14	0.9300
O4—C27	1.312 (4)	C15—C16	1.380 (4)
O4—H4	0.8200	C15—C18	1.551 (4)
N1—C8	1.330 (4)	C16—C17	1.381 (5)
N1—C9	1.332 (4)	C16—H16	0.9300
N1—Ag1^ii^	2.186 (2)	C17—H17	0.9300
N2—C3	1.330 (4)	C18—C20	1.532 (5)
N2—C2	1.338 (4)	C18—C19	1.546 (5)
C1—C2	1.365 (4)	C18—C21	1.549 (4)
C1—C5	1.383 (4)	C21—C22	1.382 (4)
C1—H1	0.9300	C21—C26	1.391 (4)
C2—H2	0.9300	C22—C23	1.381 (4)
C3—C4	1.371 (4)	C22—H22	0.9300
C3—H3	0.9300	C23—C24	1.384 (4)
C4—C5	1.388 (4)	C23—H23	0.9300
C4—H4A	0.9300	C24—C25	1.375 (4)
C5—C6	1.485 (4)	C24—C27	1.490 (4)
C6—C7	1.380 (4)	C25—C26	1.383 (4)
C6—C10	1.387 (4)	C25—H25	0.9300
C7—C8	1.373 (4)	C26—H26	0.9300
C7—H7	0.9300		
			
N2—Ag1—N1^i^	171.36 (9)	C13—C14—H14	119.4
C27—O4—H4	109.5	C14—C15—C16	118.2 (3)
C8—N1—C9	116.6 (3)	C14—C15—C18	118.9 (3)
C8—N1—Ag1^ii^	124.66 (19)	C16—C15—C18	122.8 (3)
C9—N1—Ag1^ii^	118.7 (2)	C15—C16—C17	120.5 (3)
C3—N2—C2	116.8 (3)	C15—C16—H16	119.7
C3—N2—Ag1	125.8 (2)	C17—C16—H16	119.7
C2—N2—Ag1	117.01 (19)	C12—C17—C16	121.5 (3)
C2—C1—C5	120.8 (3)	C12—C17—H17	119.2
C2—C1—H1	119.6	C16—C17—H17	119.2
C5—C1—H1	119.6	C20—C18—C19	108.8 (3)
N2—C2—C1	122.8 (3)	C20—C18—C21	106.5 (3)
N2—C2—H2	118.6	C19—C18—C21	111.8 (3)
C1—C2—H2	118.6	C20—C18—C15	113.1 (3)
N2—C3—C4	123.7 (3)	C19—C18—C15	105.4 (3)
N2—C3—H3	118.1	C21—C18—C15	111.3 (2)
C4—C3—H3	118.1	F2—C19—F1	107.3 (3)
C3—C4—C5	119.7 (3)	F2—C19—F3	107.0 (3)
C3—C4—H4A	120.1	F1—C19—F3	106.6 (3)
C5—C4—H4A	120.1	F2—C19—C18	111.2 (3)
C1—C5—C4	116.1 (3)	F1—C19—C18	112.0 (3)
C1—C5—C6	121.7 (3)	F3—C19—C18	112.4 (3)
C4—C5—C6	122.2 (3)	F4—C20—F5	106.8 (3)
C7—C6—C10	116.0 (3)	F4—C20—F6	106.5 (3)
C7—C6—C5	122.6 (3)	F5—C20—F6	106.1 (3)
C10—C6—C5	121.4 (3)	F4—C20—C18	114.1 (3)
C8—C7—C6	120.4 (3)	F5—C20—C18	111.3 (3)
C8—C7—H7	119.8	F6—C20—C18	111.6 (3)
C6—C7—H7	119.8	C22—C21—C26	118.3 (3)
N1—C8—C7	123.5 (3)	C22—C21—C18	117.8 (3)
N1—C8—H8	118.2	C26—C21—C18	123.8 (3)
C7—C8—H8	118.2	C23—C22—C21	121.3 (3)
N1—C9—C10	123.3 (3)	C23—C22—H22	119.4
N1—C9—H9	118.4	C21—C22—H22	119.4
C10—C9—H9	118.4	C22—C23—C24	120.4 (3)
C9—C10—C6	120.2 (3)	C22—C23—H23	119.8
C9—C10—H10	119.9	C24—C23—H23	119.8
C6—C10—H10	119.9	C25—C24—C23	118.3 (3)
O1—C11—O2	123.4 (3)	C25—C24—C27	119.1 (3)
O1—C11—C12	118.5 (3)	C23—C24—C27	122.6 (3)
O2—C11—C12	118.1 (3)	C24—C25—C26	121.8 (3)
C17—C12—C13	117.8 (3)	C24—C25—H25	119.1
C17—C12—C11	119.3 (3)	C26—C25—H25	119.1
C13—C12—C11	123.0 (3)	C25—C26—C21	119.9 (3)
C12—C13—C14	120.8 (3)	C25—C26—H26	120.1
C12—C13—H13	119.6	C21—C26—H26	120.1
C14—C13—H13	119.6	O3—C27—O4	123.7 (3)
C15—C14—C13	121.2 (3)	O3—C27—C24	122.8 (3)
C15—C14—H14	119.4	O4—C27—C24	113.5 (3)
			
C3—N2—C2—C1	−0.1 (5)	C16—C15—C18—C19	95.2 (4)
Ag1—N2—C2—C1	−174.0 (2)	C14—C15—C18—C21	40.0 (4)
C5—C1—C2—N2	0.2 (5)	C16—C15—C18—C21	−143.4 (3)
C2—N2—C3—C4	−0.5 (5)	C20—C18—C19—F2	−169.7 (3)
Ag1—N2—C3—C4	172.8 (2)	C21—C18—C19—F2	−52.4 (4)
N2—C3—C4—C5	1.0 (5)	C15—C18—C19—F2	68.7 (3)
C2—C1—C5—C4	0.2 (4)	C20—C18—C19—F1	70.2 (4)
C2—C1—C5—C6	−178.4 (3)	C21—C18—C19—F1	−172.5 (3)
C3—C4—C5—C1	−0.8 (4)	C15—C18—C19—F1	−51.4 (4)
C3—C4—C5—C6	177.8 (3)	C20—C18—C19—F3	−49.8 (4)
C1—C5—C6—C7	169.5 (3)	C21—C18—C19—F3	67.5 (4)
C4—C5—C6—C7	−9.0 (4)	C15—C18—C19—F3	−171.4 (3)
C1—C5—C6—C10	−8.0 (4)	C19—C18—C20—F4	−46.5 (4)
C4—C5—C6—C10	173.4 (3)	C21—C18—C20—F4	−167.1 (3)
C10—C6—C7—C8	1.3 (4)	C15—C18—C20—F4	70.3 (4)
C5—C6—C7—C8	−176.3 (3)	C19—C18—C20—F5	−167.4 (3)
C9—N1—C8—C7	−0.6 (5)	C21—C18—C20—F5	72.0 (3)
Ag1^ii^—N1—C8—C7	−179.9 (2)	C15—C18—C20—F5	−50.6 (4)
C6—C7—C8—N1	−0.3 (5)	C19—C18—C20—F6	74.3 (4)
C8—N1—C9—C10	0.5 (5)	C21—C18—C20—F6	−46.4 (4)
Ag1^ii^—N1—C9—C10	179.8 (3)	C15—C18—C20—F6	−168.9 (3)
N1—C9—C10—C6	0.6 (6)	C20—C18—C21—C22	−74.4 (4)
C7—C6—C10—C9	−1.4 (5)	C19—C18—C21—C22	166.9 (3)
C5—C6—C10—C9	176.2 (3)	C15—C18—C21—C22	49.3 (4)
O1—C11—C12—C17	−9.6 (4)	C20—C18—C21—C26	103.1 (4)
O2—C11—C12—C17	172.1 (3)	C19—C18—C21—C26	−15.6 (4)
O1—C11—C12—C13	169.8 (3)	C15—C18—C21—C26	−133.2 (3)
O2—C11—C12—C13	−8.4 (4)	C26—C21—C22—C23	−0.5 (5)
C17—C12—C13—C14	−1.1 (5)	C18—C21—C22—C23	177.1 (3)
C11—C12—C13—C14	179.4 (3)	C21—C22—C23—C24	0.5 (5)
C12—C13—C14—C15	0.5 (5)	C22—C23—C24—C25	−0.2 (5)
C13—C14—C15—C16	0.4 (5)	C22—C23—C24—C27	178.2 (3)
C13—C14—C15—C18	177.2 (3)	C23—C24—C25—C26	−0.2 (5)
C14—C15—C16—C17	−0.8 (5)	C27—C24—C25—C26	−178.6 (3)
C18—C15—C16—C17	−177.4 (3)	C24—C25—C26—C21	0.2 (5)
C13—C12—C17—C16	0.8 (5)	C22—C21—C26—C25	0.1 (5)
C11—C12—C17—C16	−179.7 (3)	C18—C21—C26—C25	−177.3 (3)
C15—C16—C17—C12	0.1 (6)	C25—C24—C27—O3	−12.0 (4)
C14—C15—C18—C20	159.8 (3)	C23—C24—C27—O3	169.6 (3)
C16—C15—C18—C20	−23.6 (5)	C25—C24—C27—O4	168.5 (3)
C14—C15—C18—C19	−81.5 (4)	C23—C24—C27—O4	−9.9 (4)

Symmetry codes: (i) *x*, *y*−1, *z*; (ii) *x*, *y*+1, *z*.

### Hydrogen-bond geometry (Å, º)

**Table:**

*D*—H···*A*	*D*—H	H···*A*	*D*···*A*	*D*—H···*A*
O4—H4···O2^iii^	0.82	1.72	2.538 (2)	174
C4—H4*A*···O1^iv^	0.93	2.52	3.366 (4)	152
C7—H7···O1^iv^	0.93	2.54	3.297 (4)	138
C8—H8···O3	0.93	2.53	3.312 (4)	142
C9—H9···O2^v^	0.93	2.48	3.242 (4)	139
C16—H16···F4	0.93	2.35	2.992 (4)	126
C26—H26···F3	0.93	2.33	2.941 (4)	123

Symmetry codes: (iii) −*x*, −*y*+2, −*z*+1; (iv) −*x*, *y*−1/2, −*z*+3/2; (v) *x*+1, *y*, *z*.
